# Recurrent *Bordetella holmesii* Bacteremia and Nasal Carriage in a Patient Receiving Rituximab

**DOI:** 10.3201/eid1910.130345

**Published:** 2013-10

**Authors:** Liem Binh Luong Nguyen, Loïc Epelboin, Jean Gabarre, Marylin Lecso, Sophie Guillot, François Bricaire, Eric Caumes, Nicole Guiso

**Affiliations:** Groupe Hospitalier Pitié-Salpêtrière, Paris, France (L.B.Luong Nguyen, L. Epelboin, J. Gabarre, M. Lecso, F. Bricaire, E. Caumes);; Université Paris, Paris (L. Epelboin, F. Bricaire, E. Caumes); and Institut Pasteur, Paris (S. Guillot, N. Guiso)

**Keywords:** Bordetella holmesii, cellulitis, pneumonia, rituximab, bacteria, respiratory infections

**To the Editor:** We report a case of recurrent *Bordetella holmesii* bacteremia with 4 clinical manifestations: 3 episodes of cellulitis and 1 episode of pneumonia. The patient, a 67-year-old man, was admitted to the Pitié-Salpêtrière hospital in Paris, France, in December 2010, for recurrent cellulitis in his left leg. Eleven years earlier, diffuse large B-cell lymphoma had been diagnosed, and he had undergone 7 chemotherapy courses. He also had received 2 autologous stem cell transplants. He was receiving maintenance treatment with intravenous (IV) rituximab every 3 months and IV immunoglobulin for hypogammaglobulinemia.

The first episode of cellulitis had occurred in his left leg 2 months before admission; the condition was treated with pristinamycin (3 g/day for 14 days), and the leg healed completely. Cellulitis recurred in his left leg 2 months later; it was again treated with pristinamycin (3 g/day) for 4 days in conjunction with fusidic acid. The cutaneous lesions worsened, and he was admitted to the hospital with fever (38.6°C) and chills.

Clinical examination showed extended cellulitis; the left leg was bright red, hot, shiny, swollen, and non-pitting. The patient’s leukocyte count was 23 × 10^9^/L (reference <10 × 10^9^/L) and C-reactive protein level was 332 mg/L (reference <5 mg/L). IV clindamycin and ceftriaxone were administered. Fever and other symptoms improved rapidly. Two consecutive blood cultures carried out before antimicrobial drug treatment were positive for *B. holmesii*, according to biochemical characteristics and molecular detection of the specific *B. holmesii rec*A gene (*1*). Isolates in both cultures were susceptible to amoxicillin, macrolide antimicrobial drugs, cefoxitin, nalidixic acid, and ciprofloxacin and were resistant to cefotaxime and trimethoprim/sulfamethoxazole (Table; blood isolate, day 1). The antimicrobial drug regimen was changed to amoxicillin (6 g/day) for 14 days; the cellulitis resolved, and the patient was discharged.

**Table:** Antimicrobial resistance profile of *Bordetella holmesii* isolates in vitro, France,, December 2010–March 2011*

Antimicrobial agent	Antimicrobial drug MICs†, μg/mL, by isolate‡				
	Blood, d 1	Blood, d 24	Blood, d 33	Blood, d 74	NPS, d 105
Amoxicillin	<2	<2	NA#	**16**	<2
Cefoxitin	<8	**>256**	**>256**	**>256**	**>256**
Cefotaxime	**>32**	**>32**	**>32**	**>32**	**>32**
Nalidixic acid	<16	<16	<16	**64**	**>256**
Trimethroprim	**>32**	**>32**	**>32**	**>32**	**>32**
Sulfamethoxazole	**>512**	**>512**	**>512**	**>512**	**>512**

*MICs corresponding to a drug resistance, which may reflect the general interpretation for nonfermenting bacteria, are in **boldface.** NPS, nasopharyngeal swab; NA, no available data. †MICs were determined by E-test on Bordet–Gengou agar. ‡Site and day (d) of collection of isolate.

Cellulitis in the right leg was diagnosed 2 weeks after the end of the previous treatment. Pristinamycin (3 g/day) was prescribed by the man’s physician but was ineffective. He was readmitted, and *B. holmesii* was again isolated in 2 new blood cultures; the organism was now resistant to cefoxitin (Table; blood isolate, day 24). Oral amoxicillin was initiated (6 g/day), without success, and after 1 week, IV ceftriaxone (2 g/day) was administered. *B. holmesii* was again isolated (isolate blood, day 33) from blood cultures despite amoxicillin treatment, and the antibiogram had the same resistance profile, except for amoxicillin (which was not determined). Because the patient was improving, IV ceftriaxone was maintained for 18 days, and he was discharged 5 days after the beginning of efficient antimicrobial drug therapy.

Two weeks after the end of the treatment, the patient was admitted to the hospital for bilateral pneumonia. Treatment with piperacillin/tazobactam and ciprofloxacin for 14 days (750 mg 2×/day) was initiated. *B. holmesii* was again isolated from blood; the bacterium had now acquired resistance to amoxicillin and nalidixic acid (Table; isolate blood, day 74). Nevertheless, ciprofloxacin treatment was continued. By real-time PCR targeting of IS*481*, *Bordetella* DNA was detected in nasopharyngeal swab (NPS) specimens (*1*), but the species could not be identified because of an insufficient amount of DNA. One month after the end of the treatment, the patient was recovering. Although the patient was asymptomatic, *B. holmesii* was isolated in a second NPS specimen. The isolate was sensitive to amoxicillin and macrolides and resistant to cefotaxime, nalidixic acid, trimethoprim, and trimethoprim/sulfamethoxazole (Table; isolate NPS, day 105). Rituximab was discontinued, and relapse had not occurred after >1 year of follow-up.

*B. holmesii* was first described in 1995 (*2*); it was primarily isolated from the blood of immunocompromised patients, especially those with spleen dysfunction. Since 1999, *B. holmesii* has been detected during pertussis outbreaks in NPS specimens of patients with pertussis-like signs and symptoms (*3*–*6*). To our knowledge, the association between *B. holmesii* infection and rituximab treatment has been reported only once, in a renal transplant recipient, and *B. holmesii* nasal carriage was not tested for (*7*).

In this patient, the *B. holmesii* infection relapses definitively stopped after rituximab treatment was interrupted, which suggests a relationship between the 2 events and that patients receiving rituximab are at increased risk for severe infection (*8*). Interpretations of antimicrobial drug resistance are difficult because no breakpoints have been defined for this species, but MICs of the drugs showed changes in the resistance profile between infectious episodes (Table). These observations strongly suggest a heterogeneous population of bacteria and that resistance was acquired after antimicrobial drug treatment as described in the United Kingdom (*9*). The patient improved while receiving ceftriaxone, although, in vitro; the bacterium was found resistant to this antimicrobial drug family as reported (*10*). Thus, the in vitro susceptibility testing and in vivo efficacy were discordant.

In conclusion, the patient’s nasal carriage and rituximab treatment may explain the recurrent infection. That the nasal carriage was the primary mode of transmission could not be proven because NPS specimens were not taken early enough. More studies are needed to determine the role of nasal carriage in *B. holmesii* bacteremia. That no *B. holmesii* infections occurred after rituximab was stopped suggests that rituximab played a role in the recurrent infections. In cases of recurrent infection or bacteremia, nasal carriage should be assessed, and the interruption of rituximab should be considered by physicians.
